# Current status and breakthroughs in treating advanced non-small cell lung cancer with EGFR exon 20 insertion mutations

**DOI:** 10.3389/fimmu.2024.1399975

**Published:** 2024-05-07

**Authors:** Meng Hu, Congying Zhong, Jiabing Wang, JinQin Chen, Tao Zhou

**Affiliations:** ^1^ Department of Oncology, Jiangxi Provincial People’s Hospital, The First Affiliated Hospital of Nanchang Medical College, Nanchang, China; ^2^ Jiangxi Medical College, Nanchang University, Nanchang, China; ^3^ Department of Chinese and Western Medicine Oncology, Jiangxi Provincial People’s Hospital, Nanchang, China

**Keywords:** non-small cell lung cancer, targeted therapy, EGFR exon 20 insertion (ex20ins) mutations, immunotherapy, new type drug EGFR-TKIs

## Abstract

Recently, targeted therapy and immunotherapy have emerged as effective treatment options for non-small cell lung cancer (NSCLC). This progress has been facilitated by the rapid development of diagnostic and therapeutic technologies and the continuous research and development of new drugs, leading to a new era in precision medicine for NSCLC. This is a breakthrough for patients with common mutations in the human epidermal growth factor receptor (EGFR) gene in NSCLC. Consequently, the use of targeted drugs has significantly improved survival. Nevertheless, certain rare genetic mutations are referred to as EGFR exon 20 insertion (ex20ins) mutations, which differ in structure from conventional EGFR gene mutations, namely, exon 19 deletion mutations (19-Del) and exon 21 point mutations. Owing to their distinct structural characteristics, patients harboring these EGFR ex20ins mutations are unresponsive to traditional tyrosine kinase inhibitor (TKI) therapy. This particular group of patients did not fall within the scope of their applicability. However, the activating A763_Y764insFQEA mutation elicits a more pronounced response than mutations in the near and far regions of the C-helix immediately following it and should, therefore, be treated differently. Currently, there is a lack of effective treatments for EGFR ex20ins mutations NSCLC. The efficacy of chemotherapy has been relatively favorable, whereas the effectiveness of immunotherapy remains ambiguous owing to inadequate clinical data. In addition, the efficacy of the first- and second-generation targeted drugs remains limited. However, third-generation and novel targeted drugs have proven to be effective. Although novel EGFR-TKIs are expected to treat EGFR ex20ins mutations in patients with NSCLC, they face many challenges. The main focus of this review is on emerging therapies that target NSCLC with EGFR ex20ins and highlight major ongoing clinical trials while also providing an overview of the associated challenges and research advancements in this area.

## 1 Introduction

According to global cancer statistics for 2020 (1), the global incidence of lung cancer ranks second, with an annual occurrence of 2.2 million new cases, representing approximately 11.4% of the total cancer incidence. Furthermore, cancer data from 2021 demonstrated that between 2014 and 2018, Lung cancer is the leading cause of death among various cancers, accounting for approximately 46% of the total cancer-related mortality rate (2). Lung cancer is one of the most threatening types of tumors to the health of human beings, and the most common histological subtype of lung cancer is non-small cell lung cancer (NSCLC), accounting for approximately 85% of all cases (3). Many patients present with advanced disease which does not allow for curative surgical and radiation therapy approaches. Consequently, it is crucial to identify effective treatment options for patients with advanced NSCLC. Molecular biology and diagnostic and therapeutic technology developments have recently been reflected in molecular-targeted therapy and immunotherapy for lung cancer. Currently, therapeutic regimens for lung cancer tend to be diverse, and the 2-year survival rate has increased to 42%. Novel therapies have improved response rates and overall survival in advanced disease with potential for long term durable remission (4, 5).

NSCLC harbors many common driver mutations, including epidermal growth factor receptor (EGFR) amplification, anaplastic lymphoma kinase (ALK) fusion, ROS proto-oncogene 1, receptor tyrosine kinase(ROS-1) fusion, BRAF V600E mutation, and Kirsten rat sarcoma viral oncogene homolog (KRAS) mutation, with EGFR mutations being the most common. NSCLC treatment has entered the era of molecular typing based on driver genes owing to the progress made in genetic testing technology (6). The data revealed that 38.1% of the patients with NSCLC in China had EGFR mutations. The EGFR mutation rate in Asian patients with lung adenocarcinoma is even higher, reaching 47.9% (7). As part of the receptor tyrosine kinase family, EGFR controls the signaling pathways that regulate cell proliferation and apoptosis (8). EGFR mutations are classified into four major categories: common EGFR mutations, T790M mutations, uncommon EGFR mutations, and EGFR ex20ins mutations (9). The EGFR gene consists of 28 exons, with most mutations occurring in exons 18–21. NSCLC with classic EGFR mutations (19 deletions and L858R) is a prominent example and accounts for 80%–90% of lung cancer driver mutations (10). These mutations are more prevalent in Asian populations, particularly among females who are non-smokers or have adenocarcinomas. Tumor cells harboring classic variants are highly sensitive to first-, second-, or third-generation EGFR tyrosine kinase inhibitors (TKIs)such as erlotinib, gefitinib, afatinib, Furmonertinib,and osimertinib,etc. (11–13). The study shows that gefitinib response rate (RR) was significantly higher in EGFR-mutant patients (37.5%) than non-mutant patients (2.6%). The phase III European Tarceva vs. Chemotherapy (EURTAC) trial also showed a significant benefit for erlotinib (9.7 months PFS) compared to chemotherapy (5.2 months PFS) in EGFR-mutant patients.Osimertinib is widely approved for the first-line treatment of patients with advanced NSCLC who have EGFR activating mutations.Cancer development relies heavily on aberrant activation of EGFR, which drives excessive cell proliferation and differentiation. Tightly regulated EGFR signaling can be disrupted by approximately 17 mechanisms. These mutations act as a “superacceptor” in asymmetric kinase dimers, favoring an active conformation (14, 15). In contrast to classical mutations, in which several EGFR mutation subtypes are sensitive to EGFR-TKIs, rare mutations, such as EGFR ex20ins mutations, are highly resistant to these drugs, the efficacy of conventional chemotherapy and targeted and immunotherapy is poor. This underscores the urgency of finding the novel therapies for these patients.

Statistics show that there are at least 102 unique ex20ins variants in the US population,withD770_N771insSVD,V769_D770insASV,A763_Y764insFEQA,H773_V774insNPH, and D770_N771insG being the most common variants (16).The development of novel targeted therapies for NSCLC patients with EGFR ex20 mutations has made remarkable progress in recent years. In 2021, the US Food and Drug Administration (FDA) approved two drugs targeting exon 20 directly: amivantamab and mobocertinib, The CHRYSALYS (NCT02609776) trial, which showed, in previously treated patients harboring exon 20 insertion mutations, a median progression free survival (PFS) of 8.3 months,40% overall response rate, 11.1 months median duration of response, The median overall survival (OS) was 22.8 months. Study 101 enrolled 114 NSCLC patients with EGFR ex20ins mutations whose disease had progressed on or after platinum-based therapy. The results showed an ORR of 28% and a median DOR of 17.5 months. Median OS was 24.0 months. Compounds such as CLN-081 and sunvosertinib/poziotinib have shown promising therapeutic effects in clinical trials and are being further validated (17), cetuximab and osimertinib or afatinib has demonstrated therapeutic efficacy in patients harboring EGFR ex20ins.Our study summarizes the current literature, conference reports, and clinical trial data on the epidemiology, clinicopathological features, and detection strategies of patients with EGFR ex20ins mutations NSCLC. The study also provides new insights and perspectives to clinical and research professionals. A review of recently developed targeted therapies for EGFR ex20ins mutations is also presented, including those approved or currently undergoing clinical trials, representing a significant challenge for clinicians.

## 2 Epidemiology and clinicopathological characteristics of EGFR ex20ins in NSCLC

EGFR ex20ins mutations have a prevalence rate of 4%–10% and slightly lower Chinese data than Western data (4.8%–5.1% vs. 9%–12%), with approximately 100,000 new patients diagnosed annually worldwide, hence, are the third most common EGFR mutations (18–20). In the NSCLC population with EGFR ex20ins mutations, Asian women younger than 60 years without a smoking history had a higher incidence of lung adenocarcinoma. This mutation type significantly reduces the drug-binding pocket size and confers increased resistance to classical EGFR inhibitors. Consequently, classical EGFR-TKI drugs are ineffective in lung cancer patients with EGFR ex20ins, and the low recurrence of EGFR ex20ins mutations.

**Figure 1:**
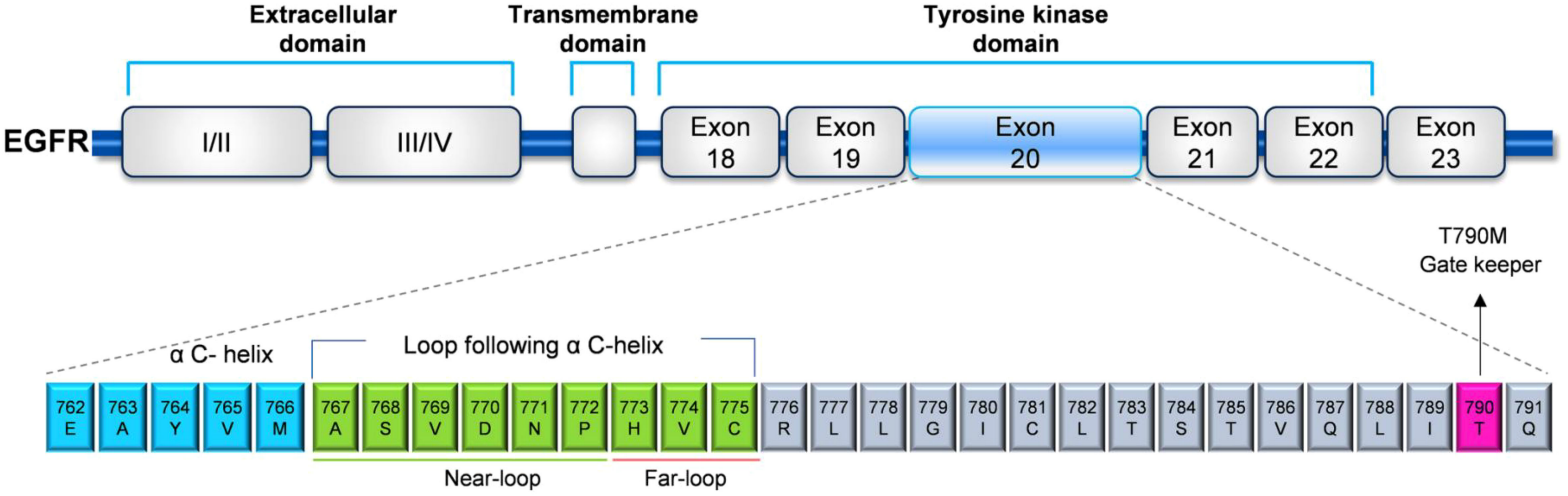
A sketch map presenting EGFR exon 20 ins. Most insertion mutations have been identified in the loop following the C-helix domain.

**Figure 2:**
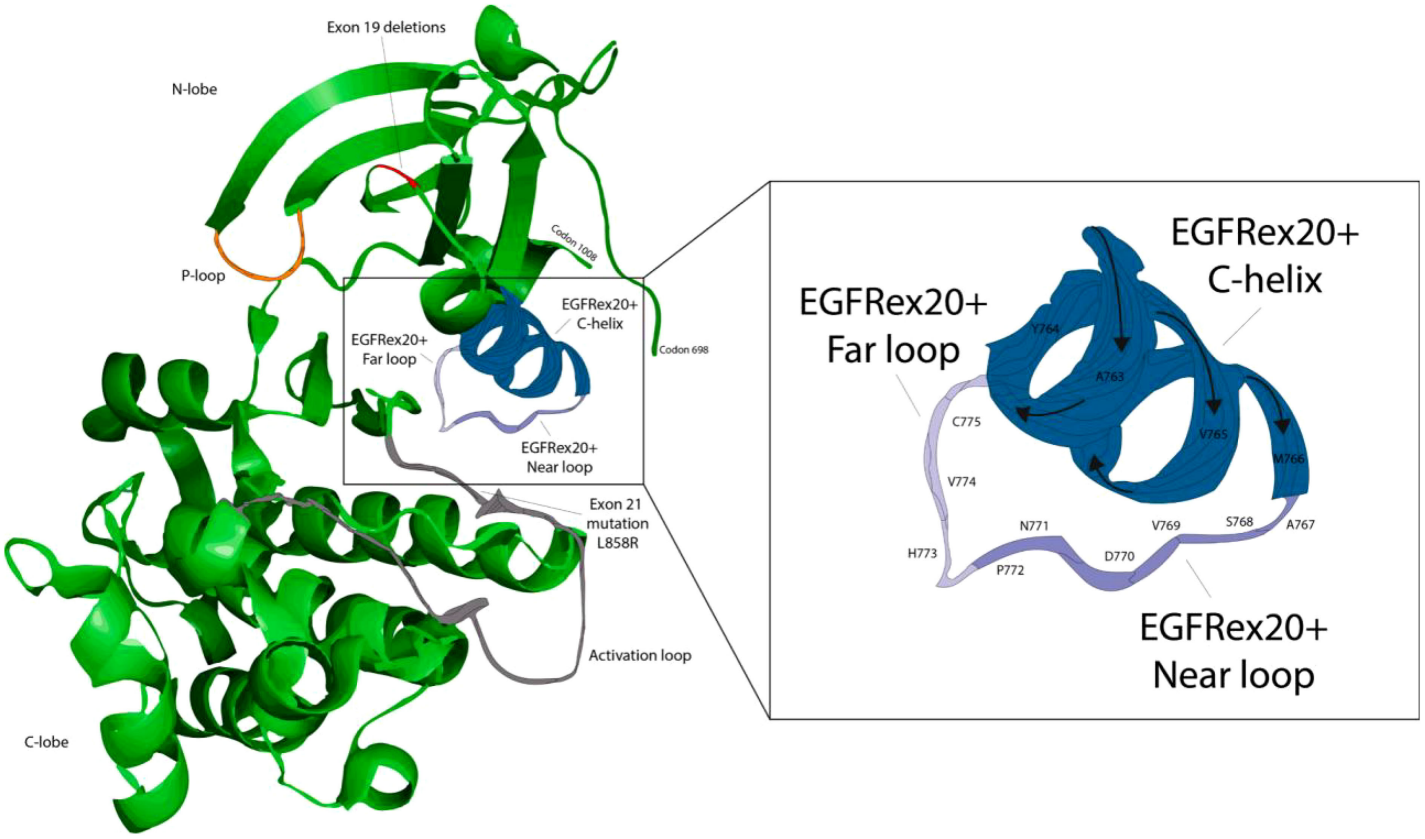
There are two lobes to the wild-type EGFR tyrosine kinase domain: the N-lobe and the C-lobe. The active and ATP-binding sites were located between the two lobes of the cleft. The location of EGFR ex20ins mutations was also highlighted. For orientation, the P loop is colored orange and the activation loop is colored gray. The image was created using PyMOl and PDB ID: 4zau (26).

EGFR ex20ins mutations contain nucleotides translated into EGFR at amino acids 762–775 of the kinase structural domain site (**Figure 1**) (21–24) and contain two major regions: the regulatory C-helix at amino acids 762-766 and a phosphate-binding loop formed by the immediately adjacent amino acids 767–774. EGFR ex20ins occur mainly in Met767–Cys775 (~90%) after the C-helix (25). Only 10% of EGFR ex20ins occur towards the C-terminal end of the C-helix (**Figure 2**) (19, 22). The mutated EGFR ex20ins NSCLC is resistant to most TKIs (e.g, gefitinib, erlotinib, and afatinib), except for the A763_Y764insFQEA mutation (22). Based on the homology model, there is a strong signal that an additional helical turn in the C helix is created by the amino acid inserted in A763_Y764insFQEA (**Figure 2**). Subsequent *in vitro* experiments demonstrated that the C-helix within EGFR interacts differently because of the additional helical turn. The modified interaction typically leads to a greater distance between the end of the beta3 strand and the beginning of the N-terminus (**Figure 1**), pulling the C-helix towards the C-in position, making it sensitive to TKIs (22, 27, 28). Currently, more than 100 activating mutations of EGFR ex20ins mutations have been identified worldwide, mainly stimulated by conformational activation of the EGFR pathway (22, 29, 30), with approximately 85 known EGFR ex20ins mutant isoforms in China (31). Common mutant isoforms include S768_D770dup, H773_V774dup, A763_Y764insFQEA, D770_N771insG, D770delinsGY, N771_H773dup, P772_H773dup, H773_V774insAH, and H773dup (32). The data (**Table 1**) suggest that the frequency of mutant subtypes in NSCLC patients harboring NSCLC patients with EGFR ex20ins mutations, with the highest frequency in A767_V769dupASV (V769_D770insASV), N771_H773dupNPH (H773_V 774i nsNPH), and S768_D770dupSVD (D770_N771insSVD) (33, 34). No crystallographic data were available for the different EGFR ex20ins subtypes. EGFR ex20ins exhibit significant heterogeneity, with extensive research and theories confirming that NSCLC with EGFR ex20ins mutations presents primary resistance to approved EGFR-TKIs. This is because it induces a rigid conformation, leading to significant spatial site block formation and small, compact drug-binding pockets. This, in turn, hinders effective EGFR-TKI binding (17, 26). Overall, we confirmed that the location of the EGFR ex20ins mutations is critical for selecting the optimal therapy. Therefore detecting all known mutations in the epidermal growth factor receptor ex20ins is important and additional research is needed”.

**Table 1:** EGFR ex20ins mutations in NSCLC: detection methods, incidence, mutation subtypes, and mutation frequency.

Detection methods	EGFR ex20ins incidence rate	Subtypes	Mutation frequency	References
NGS	Advanced NSCLC patients (IIIB-IV) 1.30%	32 subtypes A767_V769dupASV S768_D770dupSVD N771_H773dupNPH H773_V774dupHV (1)H773delinsPNPY, (2) D770delinsGY P772_H773dupPH Others	27.73% 15.97% 9.24% 5.04% each subtype was 4.20% 3.36% 30.26%	(31, 33)
NGS	2.24%	85 subtypes A767_V769dup S768_D770dup A763_Y764insFQEA, N771_H773dup, H773_V774dup D770_N771insG Others	26.51% 17.55% each subtype was 4.75% 4.39% 37.30%	(31)

NGS, next-generation sequencing.

## 3 Detection methods for ex20ins mutations

Reliable diagnosis in this patient population is critical to guide treatment because of the low incidence and high heterogeneity of EGFR ex20ins mutations. Management of NSCLC treatment involves critical genetic testing. Primary testing modalities for EGFR mutations include the amplification refractory mutation system (ARMS), Super ARMS, Sanger sequencing, immunohistochemistry (IHC), fluorescence *in situ* hybridization (FISH), digital microdot polymerase chain reaction (ddPCR), and next-generation sequencing (NGS) (35). Different genes require different detection methods. For instance, Sanger sequencing mainly designs primers for mutation sites in known disease-causing genes. Subsequently, PCR amplification and direct sequencing were used to detect known and unknown mutations. The primary gene detection methods, including NTRK gene fusion, involve FISH, RT-PCR, and NGS. PCR is suitable for relatively large tissue samples and can detect the most common genetic variations. NGS is a standard diagnostic tool for identifying the molecular heterogeneity of sequence alterations. NGS can provide faster results, and multiple genes can be simultaneously detected using a single tissue sample. For instance, NGS has detected 40–102 subtypes of EGFR ex20ins in NSCLC with higher sensitivity and precision (29, 36). The Guardant360 is an NGS-based instrument that uses cell-free DNA from plasma to identify patients with NSCLC who are likely to benefit from treatment with osimertinib. The ARMS detection method has high sensitivity and specificity, a high degree of automation, and good signal reproducibility. Although ARMS frequently detects EGFR mutations, amplification primers have been designed for known mutations. Consequently, these primers cannot be used to identify unknown mutations. IHC uses antibodies to analyze specific analytes with a high detection specificity. However, the ability to detect mutations is somewhat limited because of the availability of antibodies, and it is mainly used to detect ALK and ROS-1 gene recombination. FISH is mostly used to amplify and detect copy numbers, gene recombination, and c-MET amplification mutations. According to the current evidence-based medicine, specimen selection for analysis includes tissue specimens, pleural fluid, ascites, and sputum. Tissue specimens are the most accurate, and selecting different parts of the tissue specimens for NGS can better reflect the authenticity and accuracy of the results. Domestic and international guidelines suggest that genetic testing should range from single-gene to multi-gene testing and from single to multiple testing for the same gene. In particular, the lower sensitivity of PCR compared to NGS should be emphasized, with PCR potentially not detecting up to half of the patients with EGFR exon 20 insertion mutations. The PCR methods approved by the FDA for the assessment of EGFR mutations are Therascreen (37) and Cobas EGFRv2 (38). Therascreen can detect 3 exons using Amplified Refractory Mutation System (ARMS) PCR with sensitivities between 1% and 10%, and Cobas EGFR v2 uses the PCR-based Cobas method to detect five types of ex20ins with sensitivities ranging from 3% to 5%,We recommend second-generation sequencing for all patients with advanced lung cancer, and the likelihood of an EGFR20 insertion mutation is low that we do not recommend single-gene testing.

A significant proportion of patients with advanced NSCLC (approximately 30%) have difficulty in obtaining tissue samples acceptable for molecular analysis. Therefore, liquid biopsy using circulating tumor DNA (ctDNA) in the plasma to assess a patient’s cancer risk is significantly beneficial. Only advanced or metastatic disease is primarily recommended for liquid biopsy testing, This is because it is a measure of cell-free DNA, which is more prevalent in metastatic cancers. Therefore, they should not be used as the sole method of analysis. However, Avoiding false negatives while limiting false positives is a major challenge of liquid biopsy technology. The sensitivity of PCR-based techniques, such as qPCR or digital PCR, is significantly lower than that of NGS. In contrast, the specificity of both detection methods is 90% (39, 40).

## 4 The efficacy of conventional EGFR TKI, chemotherapy, and immunotherapy in EGFR-positive NSCLC patients

### 4.1 First− and second−generation EGFR TKIs

The exon 20ins mutations are commonly associated with resistance to first- and second-generation TKIs. In most EGFR-TKIs, mutations in ex20ins mutations lead to ineffective binding by obstructing spatial sites. Erlotinib, afatinib, and gefitinib, all examples of successful generations of EGFR-TKIs, have demonstrated significantly poor efficacy in patients with NSCLC with EGFR ex20ins mutations. For conventional treatment with EGFR-TKIs, EGFR ex20ins mutations are 100-fold less sensitive than the classical EGFR mutations (17, 22). Most patients with EGFR ex20ins mutations are resistant to first- and second-generation EGFR-TKIs, with reported response rates of 0%–27% and a median progression-free survival (PFS) of three months (41, 42). First-generation TKIs (erlotinib, gefitinib) bind reversibly to EGFR, whereas second-generation TKIs (dacomitinib, afatinib) bind irreversibly to EGFR. This study revealed that afatinib can be used as a first-line treatment for advanced NSCLC, and patients with EGFR ex20ins mutations had minimal benefit compared to patients with other mutations. However, the risk of disease progression was significantly reduced compared to standard chemotherapy, with an objective remission rate (ORR) of 23 patients with EGFR ex20ins of 8.7%, a median PFS of 2.7 months, and a median OS of 9.2 months (20).

### 4.2 Third-generation EGFR-TKIs analogs

Osimertinib is a highly irreversible and selective first-line therapy for treating patients with advanced NSCLC harboring EGFR-sensitive and EGFR T790M mutations. However, its clinical efficacy remains unclear. Osimertinib has high anti-tumor activity in two human-derived tissue xenograft models containing EGFR ex20ins (M766_A767in&ASV and H773_V774ins NPH) with a wide therapeutic window (43). Nevertheless, the efficacy of osimertinib in EGFR ex20ins mutations in lung cancer remains controversial. An ORR of 100% was achieved in a clinical trial of six NSCLC patients with EGFR ex20in mutations who were treated with osimertinib. Four of these six patients achieved partial remission (PR), Two patients achieved stable disease (SD) with an mPFS of 6.2 months and a favorable safety profile. These results provide a therapeutic option for patients with NSCLC who harbor mutations in the epidermal growth factor receptor ex20ins (44). In a real-world study (45), 62 NSCLC patients with EGFR ex20ins mutations were orally administered osimertinib 80 mg/once-daily (QD), revealing that disease Control Rate(DCR) was 53.2% with an ORR of 6.5%. However, double-dose osimertinib (160 mg/QD) (46) demonstrated a PFS of 6.8 months and an ORR of 28% in 25 NSCLC patients with advanced EGFR ex20ins mutations with common adverse events (AEs), including diarrhea, malaise, and skin dryness. Moreover, Van Veggel et al. revealed that osimertinib has limited anti-tumor activity in NSCLC with EGFR ex20ins mutations (47). Several phase II clinical studies have demonstrated the efficacy of osimertinib in patients with EGFR ex20ins NSCLC who have received prior chemotherapy or immunotherapy (44, 46). However, the efficacy of osimertinib in the treatment of NSCLC with EGFR ex20ins not uniform and requires larger clinical studies with larger samples for validation. Furmonertinib is a third-generation EGFR TKI. It was first developed in China. Furmonertinib is an oral, brain-penetrating EGFR tyrosine kinase inhibitor (TKI) with a broad spectrum of activity and selectivity for mutations, including EGFR 20ins mutations in NSCLC. A Phase Ib clinical trial of furmonertinib in EGFR exon20ins mutations NSCLC is ongoing (48). The IRC-assessed ORR for the primed furmonertinib 240 mg QD cohort was 69.0%, whereas that for the treated furmonertinib 240 mg QD and 160 mg QD cohorts was 50.0% and 40.9%, respectively. The IRC-assessed disease control rates (DCR) were 96.6%, 95.5%, and 90.9% in the furmonertinib 240 mg QD primary cohort and the furmonertinib 240 mg and 160 mg treated cohorts, respectively, with median PFS of 10.7, 7.0 and 5.8 months. The FAVOUR study showed an ORR of 38.5% versus 46.2% for double/triple dose vomitinib (160 or 240 mg/day) in patients with EGFR ex20ins mutated NSCLC (49). Irrespective of the proximal/distal cycling mutation, the anti-tumor activity of furmonertinib was observed in patients. Anti-tumor activity has also been observed in the central nervous system (CNS). This efficacy was consistent with a study on NSCLC patients with EGFR ex20ins mutations administered as second-line treatment or above (50). NSCLC patients treated with ex20ins had an overall ORR of 53.5%, a DCR of 100%, and a 3-month PFS rate of 100% in 15 patients treated with furmonertinib. A phase III, randomized, multicenter, open-label study, FURMO-004 (NCT05607550), evaluating furmonertinib versus platinum-containing chemotherapy in the first-line treatment of EGFR ex20ins mutations NSCLC is ongoing (51). We anticipate that additional large-scale clinical studies will achieve improved efficacy.

### 4.3 Immunotherapy

With the advent of precision therapy, significant progress has been made in treating lung cancer, leading to the widespread implementation of molecular-targeted and immunotherapy approaches. For patients with advanced NSCLC, molecularly targeted therapies are currently the mainstay of first-line treatment with common driver gene positivity. However, for driver gene-negative patients, chemotherapy combined with programmed cell death 1 (PD-1)/PD-ligand 1 (PD-L1) inhibitors is more effective. PD-L1 expression levels in tumor cells can predict the efficacy of immunotherapy. Meanwhile, PD-L1 expression is correlated with tumor size and brain and lymph node metastasis (52, 53). Compared with common EGFR-mutated NSCLC, PD-L1 expression in tumor tissues with rare EGFR mutations is relatively high, implying that it may benefit from immunotherapy. The study showed 76%of tumor samples were negative for PD-L1 expression, and the data suggest that the activity of immune checkpoint inhibitors (ICIs) may be impaired in NSCLC with mutations in the EGFR ex20ins (54), EGFR ex20ins mutations have reduced efficacy with ICIs (55). Although immunotherapy clinical trials have largely excluded patients with exon20ins, retrospective analyses show that patients with EGFR ex20ins appear to benefit more than patients with classic EGFR mutations. A study has shown that ex20ins mutations were associated with better response (HER-2 29%; EGFR 50%) than classic EGFR mutations (56). However, the relationship between EGFR ex20ins mutations in NSCLC and immunotherapy remains unclear (57). Moreover, some studies have indicated that PD-L1 expression levels in patients with EGFR-negative NSCLC are not correlated or predictive of immunotherapy (58). In the real world, most first-line treatments for EGFR ex20ins mutations NSCLC are platinum-containing chemotherapy regimens, and immunotherapy is primarily selected as the second-line treatment. In 2021, American Society of Clinical Oncology (ASCO) reported the first efficacy and safety data of immunotherapy in real-world NSCLC harboring EGFR ex20ins mutations, with 11 first-line patients who received immunologic monotherapy and 16 first-line patients who received immuno-combination chemotherapy having an ORR of 9.1% vs. 18.8%, median PFS of 3.1 vs. 4.5 months, and median overall survival (OS) of 11.0 vs. 11.3 months, respectively. Meanwhile, 32 patients receiving immunotherapy as a second-line treatment had an ORR of only 3.1%, median PFS of 3.3 months, and median OS of 8.1 months (59). Metro et al. (55) demonstrated that the median PFS of EGFR ex20ins mutations advanced NSCLC patients who received immunotherapy was two months. The median OS was only 5.3 months, which was deemed ineffective. Currently, immunotherapy for EGFR ex20ins mutations in NSCLC remains a significant challenge for clinical diagnosis and treatment.

### 4.4 Chemotherapy

Two-agent platinum-based chemotherapy is the standard treatment for NSCLC patients with EGFR ex20ins. The Flatiron Health Database of the U.S. Cancer Electronic Health Record in February 2020 (60) indicated that patients with EGFR ex20ins mutation-positive NSCLC, platinum-based chemotherapy regimens are the most commonly used first-line treatment. Accounting for approximately 60% of all treatments, with an ORR of approximately 20% (n = 57) and an mPFS of 4.5–5.7 months. Another study illustrated that the mPFS of EGFR ex20ins mutations NSCLC patients treated with platinum-based two-drug chemotherapy in the first-line setting was 6.4 months. In contrast, it was 4.0 months for second-line chemotherapy. These outcomes were statistically similar to wild-type patients without mutation (61). This study demonstrated that patients with EGFR ex20ins mutations NSCLC treated with pemetrexed and non-pemetrexed regimens achieved mPFS of 5.5 and 3.0 months, respectively. Furthermore, the addition of pemetrexed to chemotherapy resulted in a superior OS. Similar findings were reported in a retrospective study (62).

### 4.5 Cetuximab in combination with EGFR-TKIs

Cetuximab is an IgG1 monoclonal antibody against the EGF receptor, and the specific binding of the two blocks the intracellular activity by inhibiting tyrosine kinases that bind to the EGF receptor signaling pathway, thereby inhibiting cancer cell proliferation and inducing apoptosis. In one study, the combination of EGFR-TKIs and cetuximab completely depleted phosphorylated and total EGFR in mice suffering from L8s8R/T790M erlotinib resistance, causing near-complete tumor regression (63). Another study evaluated the efficacy of cetuximab on EGFR ex20ins through an MTS cell proliferation assay using A764 V765insHH-, A767_V769dupASV-, A763_Y764insFQEA-, and D770_N771insNPG-transduced Ba/F3 cell lines. The study displayed that cetuximab alone could not effectively inhibit the proliferation of other EGFR ex20ins mutant Ba/F3 cells, except for the EGFR-TKI-sensitive mutation A763_Y764insFQEA. Further studies have evaluated the efficacy of osimertinib or afatinib combined with cetuximab on A764_V765insHH, A767_V769dupASV, and D770_N771insNPG mutations and the efficacy of cetuximab combined with EGFR-TKIs was much better for treating EGFR ex20ins (64). A clinical retrospective report (65) studied four patients with ex20ins mutation NSCLC treated with cetuximab combined with afatinib, revealing an ORR of 75% (3/4) and mPFS of 5.4 months. These conclusions were consistent with those of previous studies, but the AEs of the combination therapy were relatively large. Therefore, the specific strategy and timing of combination therapy must be further explored.

## 5 New therapeutic strategies for patients with NSCLC who have EGFR ex20ins mutations

As previously discussed, patients with EGFR ex20ins mutations -positive NSCLC face significant barriers to receiving treatment, with minimal benefits from EGFR TKIs, chemotherapy, and immunotherapy, resulting in poor survival outcomes. Innovative targeted therapies are urgently required for these patients. The Food and Drug Administration (FDA) has approved new drugs, including mobocertinib and amivantamab, for the treatment of advanced epidermal growth factor receptor (EGFR)-expressing non-small cell lung cancer (NSCLC), with many new medicines currently in various stages of research. Clinical trials with mature data on their efficacy and safety are presented in **Table 2**. examples of Clinical Trials in patients with EGFR ex20ins mutations, for which the results are yet unavailable, are presented in **Table 3**.

**Table 2:** Examples of Clinical Trials in patients with EGFR ex20ins.

Study	Interventions	approved	N	ORR	DOR, months (95% CI)	PFS, months (95% CI)	OS, months (95% CI)	References
ECOG-ACRIN5162	Osimertinib		21	25%	5.7 (4.73– NA)	(4.07– NA)	NR	(44)
POSITION20	Osimertinib		25	28%	6.8 (4.6–9.1)	5.3 (2.7–27.6)	15.2 (14.3–16.0)	(46)
Chrysalis	Amivantamab	FDA	158	40%	11.1 months (6.9 to not reached)	8.3 (6.5–10.9)	22.8 (14.6– NR)	(66)
Study101	Mobocertinib	FDA	96	25%	5.6 months to not estimable	7.3 (5.5–9.1)	NR	(67)
The Study 101	Mobocertinib	FDA	114	28%	17.5 (7.4–20.3)	7.3 (5.5–9.2)	24 (14.6–28.8)	(68)
CLN 081	CLN-081		73	40%	40%	> 15 (estimated)	12 months	(69)
Zenith201	Poziotinib		115	14.8%	7.4	4.2	NR	(70)
WU-KONG1	Sunvozertinib	NMPA	56	50%	5.6 months for 300 mg cohort	6 months PFS for 100-, 200-, 300-, and 400-mg cohorts: 50%, 53.3%, 44.6%, and 44.4%	NR	(71)

OS, overall survival; PFS, progression-free survival; NR, not reported; ORR, overall response rate; CI, confidence interval; DoR, duration of response; EGFR, epidermal growth factor receptor.

**Table 3:** Examples of ongoing studies of patients with EGFR ex20 ins.

Trial number	Treatment plan	Phase	Primary endpoint	One line/two lines	Target number
NCT04129502	TAK-788 versus platinum-based chemotherapy	III	PFS	1	318
NCT05132777	JMT101 combined with osimertinib	II	ORR	≥2	155
NCT04036682	CLN-081	I/IIa	ORR	≥2	284
NCT05668988	Suvotinib vs. contains Platinum chemotherapy	III	PFS	1	320
NCT05607550	Vometinib vs. contains Platinum chemotherapy	III	PFS	1	375
NCT05132777	JMT101 + Osimertinib	II	ORR	≥2	155
NCT04036682	CLN-081	I/II	ORR	≥2	284
NCT20213409	BEBT-109	II	ORR	≥2	100
NCT04538664	Amivantamab and carboplatin-pemetrexed therapy vs. carboplatin-pemetrexed	III	PFS	1	300

### 5.1 New EGFR TKIs

#### 5.1.1 Mobocertinib (TAK-788 or AP32788)

TAK**-**788 is a small-molecule TKI based on the modified core backbone of osimertinib. It is a novel, highly selective oral TKI with EGFR ex20ins mutations targets (67). In 2020, the American Association for Cancer Research (AACR) reported that a phase I/II trial enrolling 28 patients with EGFR ex20ins mutations was available for evaluation. In addition to being safe and manageable, TAK-788 (160 mg QD) demonstrated an ORR of 43% and mPFS of 7 months. For patients with brain and non-brain metastasis, the ORR was 56% vs. 25%, and the mPFS was 10.2 vs. 3.7 months, indicating that mobocertinib is more effective in patients with brain metastasis (67). Therefore, the FDA has approved its use in patients with locally advanced or metastatic NSCLC with EGFR ex20ins mutations. In April 2020, the FDA recognized TAK-788 as a breakthrough therapy. Another phase I/II dose-escalation and extension study (NCT02716116) enrolled 114 NSCLC patients with EGFR ex20ins mutations who had failed platinum-based chemotherapy. The primary populations analyzed were the platinum pretreated patient (PPP) cohort and the EXCLAIM cohort, in the PPP cohort, The investigator assessment confirmed an ORR of 35%, a median PFS of 7.3 months, and a median OS of 24.0 months (68). The study reported 99% incidence of AEs in NSCLC patients with EGFR ex20in and brain metastases, including diarrhea (91%), rash (45%), and onychomycosis (38%). Furthermore, 47% of patients experienced grade 3 or higher treatment-related AEs, including diarrhea and elevated aminotransferase and aminotransferase levels. In the PPP cohort, emphasizing the importance of early detection and management of mobocertinib drug-related toxicities. The latest data from the phase I/II results of a trial (NCT02716116) presented at ASCO 2021 revealed an ORR of 28%, median PFS of 7.3 months, median OS of 24 months, DCR of 78%, and median DoR of 17.5 months. The safety profile was consistent with EGFR TKIs, with AEs in 22%–25% of patients requiring dose adjustments to reduce the dose. However, 10%–17% of patients discontinued treatment due to AE. Data from second-line therapy have assisted the introduction of mobocertinib into the Chinese market. In January 2023, mobocertinib was approved for marketing by the China National Drug Administration (NMPA) for treating patients with locally advanced or metastatic NSCLC that progressed during or after platinum-containing chemotherapy and had an EGFR ex20ins mutations. The first-line study EXCLAIM-2 (NCT04129502) of mobocertinib for treating locally advanced or metastatic EGFR ex20ins mutant NSCLC was terminated due to lack of efficacy (failure to improve PFS significantly) (72). Takeda Pharmaceutical Company initiated the voluntary delisting of mobocertinib in the United States. This suggests that less effective single agents that do not attempt combination regimens to improve efficacy are ultimately pushed out of the market. Nevertheless, mobocertinib was the first orally administered targeted therapy specifically approved for patients with advanced EGFR exon 20ins mutations-positive NSCLC. The observed response rates and durability of the responses are clinically significant and advantageous compared to existing therapies (73). Meanwhile, as of the date of this posting, no official news has been received on the withdrawal of mobocertinib from the Chinese market. Therefore, patients using this drug can continue to be used as prescribed by their doctor if they have clear indications for using the drug and have no drug resistance. Although single-agent first-line treatment clinical studies have failed, it is believed that an increasing number of clinical studies on mobocertinib combination therapy will have better efficacy and prospects in the future.

#### 5.1.2 CLN-081 (TAS6417)

It has several activities and a wider therapeutic window than most EGFR-TKIs approved or in development (74). Several EGFR ex20ins mutations are more significantly inhibited by TAS6417 than wild-type EGFR, suggesting that it is well tolerated (75). The selectivity index was much higher than that of any of the other TKIs tested. In the dose-escalation stage of the phase I/IIa study (NCT04036682), CLN-081 dose levels of 30, 45, 65, 100, and 150 mg twice daily (BID) were investigated. The efficacy expansion cohort included the 30, 65, and 100 mg dose groups. A phase IIa expansion study was initiated in the 100 mg dose group, which included 45 patients with NSCLC who had EGFR ex20ins mutations and received platinum-containing systemic chemotherapy as the first-line treatment. The results demonstrated that patients receiving long-term treatment at doses of < 150 mg BID had a tolerable safety profile, with most AEs being grade 1/2 rashes (76%), diarrhea (22%), and onychomycosis (22%), with the absence of grade ≥ 3 rashes or diarrhea. Grade 3 AEs with hepatic injury occurred in 44% of the patients, with an overall ORR of 50% (21/42) and a DCR of 64% in 42 patients whose efficacy could be assessed (67). At the ASCO meeting in June 2022, updated results from the multicenter phase I/IIa trial of CLN-081 for treating EGFR ex20ins mutations NSCLC (69). CLN-081-001 is a dose-escalation and extension trial for patients with recurrent or metastatic EGFR ex20ins mutations NSCLC who previously received platinum-containing chemotherapy. Patients with NSCLC in the groups using CLN-081 doses of ≤ 65, 100, and 150 mg had a median PFS of 8 vs. 12 vs. 8 months and median DoR of > 19 vs. > 21 vs. 7 months, respectively. The findings indicated a favorable safety profile, with rashes (74%) and diarrhea (27%) being the most common AEs. Therefore, the FDA approved CLN-081 for treating NSCLC patients with EGFR ex20ins mutations. The study is currently in phase IIa/b, and we look forward to the publication of subsequent clinical data.

#### 5.1.3 Poziotinib

Poziotinib, formerly HM781.36B, selectively interacts with EGFR/HER2/HER4 (76). *In vitro* studies demonstrated the efficacy of poziotinib in EGFR- and human epidermal growth factor receptor-2 (HER2)-dependent tumor xenograft models (17). Moreover, Cha (77) revealed that poziotinib covalently binds to EGFR and HER2 and can inhibit the classical EGFR mutation, HER2 high expression, and T790M mutation in NSCLC cell lines. The formation of ex20ins leads to changes in the C-helix conformation, forming a narrow drug-binding pocket in the ATP-binding region, which makes it difficult to interact with regular EGFR, conventional EGFR-TKIs, and NSCLC cell lines (22). poziotinib treatment in EGFR ex20ins NSCLC was associated with an ORR of 22.7% and a median OS of 9.5%. The median OS was 9.5 months, and mPFS was 5.6 months, suggesting that poziotinib effectively treats patients with EGFR ex20ins NSCLC. However, it demonstrated limited safety, and the AE incidence of grade 3 or above was 66%, with common rashes (50%) and gastrointestinal reflexes, among others. Therefore, most patients discontinued treatment, whereas others decreased their dosage (78). In contrast, poziotinib is more effective than afatinib in treating NSCLC patients with EGFR ex20ins (79). Poziotinib binds to the EGFR/HER2 kinase domain and has a smaller molecular structure than afatinib. The study also found that the sensitivity of Ba/F3 cell lines selected for EGFR ex20ins mutation was approximately 100 times greater than that of osimertinib and 40 times greater than that of afatinib (9). The ZENITH20 study (NCT03318939) was a multicenter, multi-cohort phase II clinical trial in which cohort 1 included poziotinib 16 mg QD for NSCLC patients with EGFR ex20ins and prior platinum-based chemotherapy. The median PFS was 4.2 months, ORR was 14.8%, DCR was 68.7%, and the median DoR was 7.4 months. However, 88% of the patients discontinued their medication because of AEs, 68% reduced their dosage, and 10% permanently discontinued it (80). Cohort 3 consisted of patients with advanced or metastatic NSCLC treated with EGFR ex20ins in the first-line setting. A total of 79 subjects were enrolled, with a ORR of 27.8%, median PFS of 7.2 months, DCR of 86.1%, and median DoR of 6.5 months (81). The cohort in the ZENITH20-5 study used a 16 mg QD or 8 mg BID comparison regimen, revealing that the ORR of 31.6% and DCR of 78.4% in the 8 mg BID group (n = 19) were significantly higher than those of 15.8% and 52.6% in the 16 mg QD group (n = 19). The proportion of serious AEs was significantly lower (23% vs. 35%). A phase II clinical study (NCT03066206) for treating EGFR ex20ins-mutated NSCLC demonstrated that the ORR was 58%, the mPFS was 5.6 months, and the DCR was 90% (80). Combined efficacy and safety data have led to the voluntary termination of clinical registration studies on poziotinib in China. By the end of 2022, the FDA rejected the marketing application of poziotinib; therefore, further exploratory clinical studies are in progress.

#### 5.1.4 DZD9008 (Sunvozertinib)

DZD9008, a highly selective and irreversible EGFR-TKI designed to target EGFR/HER2 ex20ins, was developed by Dizhe Pharmaceuticals to target various subtypes of EGFR. DZD9008 is the first original Chinese Class I drug targeting EGFR ex20ins in advanced NSCLC with lower affinity for EGFR wild-type (70) because of its molecular structure containing an acrylamide moiety and an amino-pyrimidine parent ring. Therefore, adding an aniline structure at the C-4 position of the pyrimidine parent ring makes the molecular structure more flexible, increasing its inhibitory effect on EGFR ex20ins. Amino-pyrimidine effectively inhibited EGFR-sensitive and T790M drug-resistant mutations. The selectivity of Sunvozertinib for EGFR ex20ins was 3–10 times higher than that of the wild-type. Sunvozertinib inhibited EGFR ex20ins at half the inhibitory concentration (50%) in tumor cell lines expressing EGFR L8s8R, Exonl9del, L858R/T790M, and multiple ex20ins. The study of DZD9008 by Yang et al. (82, 83) demonstrated that NSCLC patients with EGFR ex20ins had an ORR of > 40%, and the ORR for intravenous amivantamab (JNJ) was > 40%. The study also revealed that the ORR of EGFR ex20ins NSCLC patients treated with 300 mg QD of DZD9008 was 41.9%, whereas those treated with 200 mg QD of DZD9008 had an ORR of 45.9%. This indicates that low-dose DZD9008 was effective. In December 2020, DZD9008 was added to the NMPA’s list of “breakthrough” drugs for treating locally advanced or metastatic NSCLC harboring an EGFR ex20ins in patients who have previously received chemotherapy. At the 2021 ASCO annual meeting, data were collected from the DZD9008 phase I clinical study efficacy assessment cohort (56 patients, EGFR ex20ins) and the safety assessment cohort (102 patients, EGFR or HER2 mutations). In particular, 31 patients in the efficacy assessment cohort treated with 300 mg QD of DZD9008 had a DCR of 90.3%, an ORR of 41.9%, and an incidence of treatment-emergent AEs of grade 3 or higher (33.3%). Among the 11 patients receiving 200 mg QD, the DCR was 81.8%, ORR was 45.5%, and the incidence of grade 3 or higher treatment-related AEs was 6.3% (70). The findings of two ongoing phase I/II studies (WK-KONG1 and WUKONG2) were reported in ASCO 2022 (84), which targeted patients who had previously received platinum-based chemotherapy with DZD9008 at a dose of 50–400 mg QD administered to patients with EGFR ex20ins mutations who experienced disease progression after previous treatment with platinum-based chemotherapy and PD-L1. The results revealed that the ORR values for the 100, 200, 300, and 400 mg cohorts were 50.0%, 55.6%, 44.8%, and 22.2%, respectively, whereas the 6-month PFS rates were 50.0%, 53.3%, 44.6%, and 44.4%, respectively. These findings indicate that DZD9008 was well tolerated, with diarrhea and rash being the most common on-treatment AEs. The European Society for Medical Oncology(ESMO) Congress 2022 presented a multicenter phase II WU-KONG6 study (85), confirming that the 300 mg dose ORR was 59.8%, and the ORR in patients with brain metastases at baseline was 48.4%. The ORR of sunvozertinib in patients with different EGFR ex20ins subtypes was 62% (proximal loop end insertion mutation) and 50% (distal loop end insertion mutation). On July 31, 2022, four global sunvozertinib studies included 277 patients with advanced EGFR- or HER2-mutated NSCLC in the safety analysis set (70), demonstrating that sunvozertinib had an excellent overall safety profile with common AEs similar to those of conventional EGFR-TKIs, including rash and diarrhea. With superior efficacy and safety, sunvozertinib was granted a Breakthrough Therapy Designation (BTD) in China and the U.S. in 2020 and 2022, respectively. Moreover, the first indication of sunvozertinib for treating NSCLC with EGFR ex20ins when standard therapies are ineffective was included in the National Medical Products Administration (NMPA) priority review in January 2023. The results of the first pivotal study of sunvozertinib (WU-KONG6) were presented at the 2023 ASCO annual meeting. The Independent Imaging Review Committee (IRC) confirmed an ORR of 60.8%. More than 90% of patients experienced target lesion shrinkage with sunvozertinib monotherapy, with a DCR of 87.6%. Sunvozertinib is now approved in China as a targeted drug for treating EGFR exon20ins mutations advanced NSCLC. The remission rate for second/postline treatment is promising, with an ORR of 61%, more than 50%, and a high DCR. The median PFS was 6.5 months, with an acceptable safety profile. The adverse reactions were similar to those of conventional EGFR-TKIs and mainly were grade 1–2, clinically manageable, and reversible. Another domestic and international phase I/II study demonstrated that the optimal ORR reached 77.8% when treating advanced NSCLC with EGFR ex20ins using first-line single-agent RP2D dosing (300 mg QD) of sunvozertinib (85). An ongoing global multicenter phase III study compared sunvozertinib with platinum-containing chemotherapy as the first-line treatment for EGFR ex20ins mutations NSCLC (WU-KONG28) to confirm its excellent efficacy. In 2023, the ESMO published preliminary results of first-line therapy with sunvozertinib in patients with EGFR ex20ins mutant advanced NSCLC, indicating an encouraging ORR of 78.6% for first-line treatment with sunvozertinib monotherapy exceeding 70%, and a median PFS of more than one year. We look forward to the presentation of better clinical data and early benefits for global patients.

### 5.2 Response to novel antibodies

#### 5.2.1 Amivantamab

Amivantamab is a fully humanized, bispecific immunoglobulin G1 (IgG1)-based antibody. It exerts its anti-tumor effects by binding to EGFR and c-MET (56) on the tumor surface, thereby inhibiting the activation of their signaling pathways and binding to extracellular segments to promote receptor-antibody complex degradation (71). Amivantamab in 15 NSCLC patients with EGFR ex20ins demonstrated promising clinical anti-tumor activity (86). A Phase I/II, open-label, multicenter, dose-escalation, multi-cohort study (CHRYSALIS) revealed that amivantamab has effective and safe therapeutic potential in EGFR ex20ins NSCLC (66). The preliminary findings of EGFR ex20ins in the arm (Cohort D) of the II phase of the CHRYSALIS study were reported during the 2020 ASCO meeting. The results revealed that after amivantamab treatment, 39 evaluable patients had an ORR of approximately 40%, DoR of 10 months, OS of 22.8 months, and median PFS of 8.3 months. The common AEs were rash, infusion-related reactions, onychomycosis, and interstitial lung disease. The most common grade 3–4 AEs were hypokalemia (5%), pulmonary embolism (4%), diarrhea (4%), neutropenia (4%), and rash (4%) (87). The FDA first approved the marketing of this drug on May 21, 2021, primarily for patients with EGFR ex20ins mutations who were treated with platinum-based drugs and mutations in adult patients with locally advanced or metastatic NSCLC refractory to platinum therapy (88). A recent ASCO (2022) study demonstrated that mobocertinib and amivantamab had similar efficacy in NSCLC patients with EGFR ex20ins who had disease progression during or after platinum-based chemotherapy. In the first-line study, PAPILLON (NCT04538664) (89), an international, multicenter, randomized, open-label, Phase III clinical trial, 308 patients were randomized (153 to amivantamab -chemotherapy and 155 to chemotherapy alone). The findings indicated that the amivantamab chemotherapy group achieved a significantly longer PFS than the chemotherapy group (median 11.4 vs. 6.7 months; hazard ratio for disease progression or death 0.40). At 18 months, PFS was achieved in 31% of the patients in the amivantamab chemotherapy group compared to 3% in the chemotherapy group. Consequently, amivantamab, in combination with chemotherapy (ACP regimen), prolonged the median PFS from 6.7 to 11.4 months, improved the ORR from 47% to 73%, and achieved efficacy data in Asian patient populations consistent with global populations. The 2024 V1 edition of the NCCN guidelines recommends the ACP regimen as the first-line treatment for EGFR exon 20 insertion mutations (class 1 evidence).

### 5.3 HSP-90 inhibitor

#### 5.3.1 Luminespib

Luminespib is designed to inhibit and prevent Heat Shock Proteins (HSP90). HSP90 is a molecular chaperone responsible for the maintenance and stable improvement of protein folding, which promotes the stabilization and activation of various client proteins, tumorigenicity, and value addition (90). HSP blockers degrade EGFR ex20ins and their downstream signaling pathways, thereby inducing apoptosis. A phase II, single-arm, open clinical study (NCT01854034) included 29 patients with stage IV EGFR ex20ins-mutated NSCLC who received prior platinum-containing chemotherapy. The outcomes revealed an ORR of 17%, representing a significant benefit compared to afatinib. However, the Poor PFS and OS data, which is not clinically advantageous (91). Common AEs include diarrhea, ocular toxicity, hypertension, fatigue, and visual changes. Further clinical studies are required to explore its efficacy.

### 5.4 Other drugs

Numerous drugs such as tarloxotinib (92), JS 111 (AP-L1898), pyrrolizidine, BDTX-189, JMT101, alflutinib (93) (AST2818/Furmonertinib), and BLU-451 are in early clinical studies and are the focus of further clinical investigations and ongoing trials in patients with EGFR ex20ins.

## 6 Treatment strategies for early and locally advanced NSCLC with EGFR exon 20 insertion mutations

Currently, the therapeutic strategy for early and locally advanced NSCLC with exon 20 insertion mutations is similar to that for driver gene-negative NSCLC, but research comparing adjuvant and neoadjuvant therapies is lacking. Surgery is the best option for patients with early-stage NSCLC. However, the rate of local and metastatic recurrence is high after surgery alone, particularly in patients with NSCLC and stage III N2 lymph node involvement. Published studies have revealed the benefits of immunotherapy and targeted therapy in adjuvant and neoadjuvant settings, and many additional studies are ongoing.

### 6.1 Operable lung cancer

#### 6.1.1 Adjuvant therapy

The five-year survival rate of patients with early-stage NSCLC following complete resection remains highly variable, with a significantly higher risk of recurrence and metastasis. Statistical analysis of 94,703 cases of NSCLC classified by the AJCC 8th edition tumor staging revealed that the five-year survival rates were 90%, 73%, 65%, and 41% for patients with pathologic stages IA1, IB, IIA, and IIIA, respectively. In addition, the Chinese Society of Clinical Oncology (CSCO) guidelines estimate that approximately 20%–40% of stage I–II NSCLC patients will develop local recurrence or distant metastasis after complete resection. Surgical resection is the primary treatment modality for early-stage NSCLC. However, after complete resection, local recurrence or distant metastasis occurs in about 20%–40% of patients with stage I–II NSCLC. Platinum-based chemotherapy with a two-drug regimen has become the standard postoperative treatment for resectable stage II–III NSCLC.

The IMpower010 trial (94) achieved primary endpoint with a statistically significant improvement in three-year DFS from 48% to 60% in patients with PD-L1 > 1%. Three-year DFS improved from 49% to 56% in the overall stage II to IIIA population. Subgroup analysis revealed a graded increase in benefits with increasing levels of PD-L1 expression, without difference in outcomes observed based on EGFR or ALK mutations. This study led to the FDA approval of adjuvant atezolizumab for one year after surgical resection of stage IB–IIIA NSCLC (95). Subsequently, the European Commission approved atezolizumab as an adjuvant therapy for NSCLC following complete resection and platinum-based chemotherapy for treating adult patients with NSCLC whose tumors do not harbor EGFR or ALK mutations and have PD-L1 expression of at least 50% (96). The median DFS in the Phase III PEARLS/Keynote 091 trial was not achieved at 35.6 months of follow-up (97). This suggests that combination immunotherapy is promising for adjuvant therapy, and it is believed that further clinical trials will yield encouraging data, there are ongoing studies in the EGFR exon 20 insertion mutations.

#### 6.1.2 Neoadjuvant therapy

The study indicated that chemotherapy works synergistically with the immune system (98), and PD-L1 expression in NSCLC is stimulated by chemotherapy (99–101). The study demonstrated that in resectable NSCLC, atezolizumab plus carboplatin and nab-paclitaxel resulted in an MPR of 57%, including pCR, in 33% of the 30 patients (102). In a phase II trial, neoadjuvant nivolumab plus carboplatin and paclitaxel-induced major pathologies in remission in 34 of 41 patients with resected stage IIIA NSCLC, with an 18-month OS of 93.5% (103). Comparable pathological remission rates were observed in the single-arm SAKK trial in patients with N2 and IIIA NSCLC (104). These findings have led to a number of clinical trials of neoadjuvant chemotherapy in combination with immunotherapy, and we expect to obtain pathologic endpoints from some of these trials within the next year (105). The Checkmate 816 (106), NADIM II (103), and CTONG1804 (107) trials suggested that neoadjuvant immunotherapy + chemotherapy is a better treatment option for operable NSCLC.

DS-8201 has demonstrated an ORR of 55% in previous studies for metastatic HER2-mutated NSCLC. However, the efficacy and safety of DS-8201 have not been evaluated in early and locally advanced NSCLC. Prof. Wenzhao Zhong et al. published the efficacy and safety of neoadjuvant DS-8201 treatment (three cycles, 345 mg, Q3W) in one patient with stage IIIA NSCLC harboring EGFR 20 exon insertion mutations after neoadjuvant therapy with sequential R0 surgical resection. DS-8201 demonstrated anti-tumor activity in patients with locally advanced NSCLC with a low incidence of AEs, and neoadjuvant therapy did not delay surgical treatment. DS-8201 had superior efficacy regardless of HER2 protein expression. This case provides a new idea for using antibody-drug conjugate(ADC) drugs in neoadjuvant therapy.

### 6.2 Unresectable or inoperable disease

Definitive stereotactic body radiotherapy is important treatment for most patients with medically inoperable stage I and II node-negative NSCLC, with a biologically effective dose ≥ 100 Gy (108). However, The patients with large primary tumors, central tumors, etc. may not be eligible for SBRT.Long-term local tumor control rates of 90%–95% with this approach and PFS of 70%–80% for metastatic and systemic diseases (109–111). It is difficult to treat patients with systemic drug therapy because they are frail, elderly, and have many underlying diseases that preclude surgical treatment (112, 113).

Approximately 25% of patients diagnosed at the first visit had unresectable stage IIIA–IIIC NSCLC. Based on the results of PACIFIC, the standard of care for these patients has been the addition of immunosuppressive therapy after concurrent radiotherapy (114). The primary treatment for patients with unresectable locally advanced, node-positive (stage IIB–IIIC) NSCLC is definitive concurrent chemotherapy and radiation followed by one year of durvalumab treatment. Clinical practice involves the use of sequential radiation therapy as an alternative for most Chinese patients who are unable to tolerate the toxicity of concurrent radiotherapy. However, there is still insufficient data from large clinical trials to confirm the efficacy of this therapy. The GEMSTONE⁃301 study is an evaluation of the efficacy and safety of sugemalimab, as consolidation therapy in patients with unresectable stage III NSCLC who have not experienced disease progression after concurrent or sequential radiotherapy (115). The results of this study demonstrated clinical benefits after both concurrent and sequential radiotherapy, confirming that immunoconsolidation therapy following sequential radiotherapy is feasible and fills a gap in the standard of care for unresectable stage III NSCLC, there are ongoing studies in the EGFR exon 20 insertion mutations.

## 7 Future perspectives and conclusions

Among patients with EGFR mutations, EGFR ex20ins frequency is rare and has multiple subtypes. The unique molecular structure of EGFR ex20ins leads to the poor efficacy of traditional TKI treatment and limits clinical therapeutic approaches. Currently, the first-line treatment modality is chemotherapy combined with immunotherapy. As new drug development progresses and advances, the biological characteristics and treatment options of NSCLC are better understood owing to the rapid growth of precision-targeted therapy. Studies have revealed that the therapeutic effect of novel TKI drugs targeting EGFR ex20ins can achieve better clinical outcomes (116). The FDA has approved mobocertinib with amivantamab for patients with EGFR ex20ins mutations NSCLC who have undergone platinum-based chemotherapy failure or progression. Furthermore, guidelines recently recommended mobocertinib. To further improve the level of precise diagnosis and individualized treatment of NSCLC, circulating tumor DNA (ctDNA) is used to dynamically monitor changes in tumor molecular markers during targeted therapy in patients with EGFR ex20ins, mainly to explore emerging drug-resistance-related genetic variants, which will be an important future direction.

Domestic and international guidelines recommend routine EGFR ex20ins gene testing for advanced NSCLC with adenocarcinoma components (117, 118). Consequently, the importance of targeting EGFR ex20ins testing needs to be increased. Real-time fluorescence quantitative PCR is a commonly used detection technique suitable for clinical implementation because of its relatively high sensitivity and ease of operation. However, EGFR ex20ins are more heterogeneous, and PCR covers a limited number of subtypes. Therefore, this may lead to failure in detecting patient mutations during clinical applications. Moreover, 40%–50% of patients do not exhibit detectable levels of this mutation (119). The NGS assay has high throughput and sensitivity, can detect unknown mutation sites, and covers all EGFR ex20ins sites. Theoretically, it can detect all the EGFR ex20ins subtypes. However, NGS testing has high sample requirements and complicated operation steps, is relatively expensive, and is time-consuming. Therefore, the extensive application of NGS in clinical settings remains a challenge. Introducing new targeted drugs has brought hope for treating advanced NSCLC with rare mutations, which remains challenging and requires further exploration. First, it is imperative to improve the quality and selectivity of genetic testing. Second, the resistance mechanisms of new targeted drugs remain unclear. Third, it is necessary to understand the gene mutation spectrum of rare mutation NSCLC further, and it is necessary to understand whether some subclonal genes lead to drug resistance. Fourth, the optimal combination of targeted therapy, chemotherapy, anti-angiogenic therapy, and immunotherapy must be explored to further improve the overall efficacy.

In conclusion, lung cancer treatment has evolved into a diverse and precise approach. One of the most challenging research areas is the treatment of EGFR ex20ins mutant NSCLC, which has become a prominent focus. The efficacy of newly developed targeted drugs for this condition has been observed to a certain extent. Significant progress has been made recently, and the NCCN-recommended drugs, mobocertinib and amivantamab are at the forefront of targeted therapies for this patient population (120). The mechanisms of action and routes of administration of these two drugs are different, presenting additional treatment options for patients. Moreover, there are now some promising targeted treatments in the development pipeline, potentially boosting clinical outcomes for this subset of patients. However, it is important to acknowledge the numerous challenges, indicating a long and arduous path ahead. We are confident that a transition to a new era of precision-targeted therapy will soon occur.

## Author contributions

MH: Investigation, Resources, Supervision, Validation, Visualization, Writing – original draft, Writing – review & editing. CZ: Data curation, Writing – review & editing. JW: Data curation, Formal Analysis, Writing – original draft. JC: Investigation, Writing – original draft. TZ: Formal Analysis, Writing – original draft.
